# Genome-wide association study for loin muscle area of commercial crossbred pigs

**DOI:** 10.5713/ab.22.0407

**Published:** 2023-01-11

**Authors:** Menghao Luan, Donglin Ruan, Yibin Qiu, Yong Ye, Shenping Zhou, Jifei Yang, Ying Sun, Fucai Ma, Zhenfang Wu, Jie Yang, Ming Yang, Enqin Zheng, Gengyuan Cai, Sixiu Huang

**Affiliations:** 1 College of Animal Science and National Engineering Research Center for Breeding Swine Industry, South China Agricultural University, Guangzhou, Guangdong 510642, China; 2 Guangdong Zhongxin Breeding Technology Co., Ltd, Guangzhou, Guangdong 510642, China; 3 Guangdong Provincial Key Laboratory of Agro-animal Genomics and Molecular Breeding, South China Agricultural University, Guangzhou, Guangdong 510642, China; 4 Yunfu Subcenter of Guangdong Laboratory for Lingnan Modern Agriculture, Yunfu, Guangdong 527300, China; 5 College of Animal Science and Technology, Zhongkai University of Agriculture and Engineering, Guangzhou, Guangdong 510642, China

**Keywords:** Genome-wide Association Study (GWAS), Loin Muscle Area, Pig, Single Nucleotide Polymorphism

## Abstract

**Objective:**

Loin muscle area (LMA) is an important target trait of pig breeding. This study aimed to identify single nucleotide polymorphisms (SNPs) and genes associated with LMA in the Duroc×(Landrace×Yorkshire) crossbred pigs (DLY).

**Methods:**

A genome-wide association study was performed using the Illumina 50K chip to map the genetic marker and genes associated with LMA in 511 DLY pigs (255 boars and 256 sows).

**Results:**

After quality control, we detected 35,426 SNPs, including six SNPs significantly associated with LMA in pigs, with MARC0094338 and ASGA0072817 being the two key SNPs responsible for 1.77% and 2.48% of the phenotypic variance of LMA, respectively. Based on previous research, we determined two candidate genes (growth hormone receptor [*GHR*] and 3-oxoacid Co A-transferase 1 [*OXCT1*]) that are associated with fat deposition and muscle growth and found further additional genes (*MYOCD*, *ARHGAP44*, *ELAC2*, *MAP2K4*, *FBXO4*, *FBLL1*, *RARS1*, *SLIT3*, and *RANK3*) that are presumed to have an effect on LMA.

**Conclusion:**

This study contributes to the identification of the mutation that underlies quantitative trait loci associated with LMA and to future pig breeding programs based on marker-assisted selection. Further studies are needed to elucidate the role of the identified candidate genes in the physiological processes involved in LMA regulation.

## INTRODUCTION

Pork is an important source of animal protein for humans, and the lean meat percentage is the most important breeding goal in important economic traits [1]. The loin muscle area (LMA), which is referred for the measurement of the large muscles on the back of the pig, also mainly constitutes the meat in the pork chop [2]. The LMA has a strong genetic correlation with lean meat percentage and is therefore valuable for predicting lean meat percentage [3]. The LMA is also helpful in predicting carcass traits, artificial selection, and growth experiments [4]. The key factors affecting psoas muscle growth mainly include nutrition (such as the protein content of the diet), management (such as the environment of the pig farm), and genetics. According to previous studies, the heritability of LMA is estimated to be between 0.35 to 0.47 [5–7], which is a moderately heritable trait and can be genetically improved.

In actual production, the most common way to measure LMA is through an A scan, however due to the error of the machine itself and other reasons it is difficult to get an accurate measurement [8]. Subsequent slaughter measurements of the LMA greatly reduces the error. But difficulties in phenotypic measurement still hinder the selection of LMA. With the advent of molecular breeding, in order to dissect the genetic molecular mechanisms of LMA, researchers have used different strategies to study different populations; for instance, the exploration of microsatellite markers in the pig genome and genome scans to detect quantitative trait loci (QTLs) in LMA [9] or using molecular genome scan analysis to identify chromosomal regions influencing LMA in pigs [9]. In the pig QTLs database [10] (Release 48, Aug 24, 2022), a total of 35,846 QTLs are identified, of which 416 are associated with LMA (1.16% of 35,846 QTLs), but the number of QTLs is still relatively low. QTL mapping throughout the genome for linkage mapping is normally difficult due to the low marker density, and most QTLs are usually maintained at large confidence intervals (over 10 Mb, including hundreds of genes), which severely hinders the optimization of plausible candidate genes [11]. Despite some progress in traditional genetic improvement of carcass and growth traits, elucidation of the biological mechanisms of LMA is still difficult and challenging [12].

With the rapid advancement of the livestock and poultry genome project, high-throughput pig single nucleotide polymorphism (SNP) chips were commercialized, which could effectively improve the accuracy of gene mapping in the pig genome [13]. Genome-wide association analysis (GWAS) has been widely used in humans as a powerful strategy to detect genetic variants associated with complex traits. In the studies on pigs, GWAS has gradually become an indispensable tool for exploring the genetic variation and structure of major economically advantageous traits in pigs [14–16]. GWAS was also used to detect the underlying genetic mechanism for LMA. He et al [17] used the Laiwu pig population for GWAS analysis and identified three candidate genes (far upstream element binding protein 3 [*FUBP3*], myosin heavy chain [*MYH*] family, leucine-rich repeats and guanylate kinase domain containing [*LRGUK*]). Ding et al [18] performed a genome-wide analysis for copy number variation (CNV) detection using GeneSeek Porcine SNP50 Bead chip data of 3,770 pigs and identified one CNV region (CNVR) associated with LMA. Fan et al [19], used the IIuminaPorcineSNP60K chip to perform GWAS analysis on 820 female commercial pigs from Large White×Landrace cross and found two significant SNPs, *M1GA0002180* and *M1GA0002244*, and identified the insulin-like growth factor 2 (*IGF2*) gene as a candidate gene. Zhuang et al [20] performed meta-GWAS of 6043 Duroc pigs for LMA and loin muscle depth traits and identified eight potential candidate genes. However, although previous studies have been helpful to LMA research, the key genes that influence LMA are yet to be identified, and the results of GWAS on LMA of pigs are inconsistent; consequently, the genetic mechanism of LMA requires further study. Furthermore, as a favorite source of pork, research on the LMA of DLY pigs is even more necessary. Therefore, we used LMA measurements of 511 DLY slaughtered pigs to perform GWAS to find important molecular markers and candidate genes for LMA. We hoped to provide a theoretical basis to explain the genetic basis of LMA and the development of molecular breeding, as well as provide biological guidance for subsequent breeding practices.

## MATERIALS AND METHODS

### Ethics approval

Treatment of all animals used in this study was as per the guidelines for the care and use of experimental animals established by the Ministry of Agriculture of China. Tissue samples from pigs were collected after obtaining approval from the ethics committee of South China Agricultural University (Guangzhou, China; approval no 2018F098).

### Experimental animals

In this study, 511 DLY pigs (255 boars and 256 sows) raised under the same rearing standards were obtained from Wen’s Food Group Co., Ltd (Guangdong, China). Measurement of the LMA was carried out after slaughter. Specifically, the longissimus dorsi muscle was sampled at the last rib, and a sulfuric acid paper was used to draw the eye muscle area, and then a planimeter or a square grid was used. The area drawn on the sulfate paper was measured to determine LMA.

### Genotyping and quality control

DNA was extracted from pig ear tissue using the phenol-chloroform method. DNA quality was measured by electrophoresis, and the absorption ratio (A260/280) was estimated. All quality DNA samples were diluted to 50 ng/μL, as described by Ding et al [21]. Genotyping was performed using GeneSeek porcine 50 K SNP chip from Illumina, then PLINKv1.09 [22] software was used for data editing. The quality control (QC) conditions for individuals were SNP markers call rate <95%, the QC conditions for SNPs were: minor allele frequencies <1%, and Hardy–Weinberg test p<10^−06^ were excluded. Unmapped SNPs and SNPs located on sex chromosomes were removed. Finally, after QC, a total of 35,426 SNPs were used for subsequent GWAS on LMA.

### Single-locus genome-wide association study

The GWAS was performed using the method proposed by Zhou and Stephens [23]. To correct the population structure, an efficient mixed linear model was used to analyze the effect of a single SNP on phenotypic traits to develop the genetic algorithm [24]. GEMMA v0.94.1 software was used for association analysis of the characteristics [23], and the statistical formula is as follows:


y=Wα+xβ+μ+ɛu~MVN n (0,λτ^(-1) K),ɛ~MVN n (0,τ^(-1)In

where *y* is the vector of phenotypic values for LMA for all individuals and W is the correlation matrix based on appropriate fixed effects. To ensure that we could choose suitable fixed effects, we used EIGENSTRAT [25] to test the significance of the top five principal components (PCs) as covariables, and conducted analysis of variance by R language for the fixed effects of the selection. According to the test results (Supplementary Table S1), we finally determined five eigenvectors of principal components, slaughter batch and live weight were included as fixed affects. α is the vector of the corresponding coefficients, including the intercept, x is the vector of SNP marker genotypes, β is the corresponding effective vector of SNP markers, u and ɛ are vectors of random effects and random errors, respectively, τ^(−1) is the variance of the random errors. λ is the ratio between the two variance components, K is the standard correlation matrix of software estimation, *I* is an identity matrix, and MVN is a multivariate normal distribution. The heritability of LMA was estimated by the genome-wide complex trait analysis (GCTA) software.

### Identification of significant single-nucleotide polymorphisms associated with loin muscle area

Significant SNPs were identified for LMA as those that surpassed the threshold with a false discovery rate (FDR) controlled at 0.01 [26]. The threshold p-value was estimated as follows:


p=FDR×n/m

where n represents the number of SNPs with p<0.01 in the GWAS results, ordered ascendingly by their effects, and m is the number of analyzed SNPs. In brief, the final selected threshold was 1.13×10^−04^, and the obtained GWAS results were visualized using R language [27]. The phenotypic variance explained by each significant SNP was estimated by the GCTA v1.94 software with “--reml” parameter [28,29].

Candidate genes 0.5 Mb upstream and downstream of significant SNPs were screened, and the functional genes were annotated on the Sus scrofa 11.1 genome from Ensemble genome database v107 (http://asia.ensembl.org/Susscrofa/Info/Index). The significant annotated genes were selected to conduct Kyoto encyclopedia of genes and genomes (KEGG) pathways and gene ontology (GO) analyses by using the KOBAS3.0 database [30]. The significance of the enriched terms was assessed using Fisher’s exact test, with p<0.05, and the genes involved in biological processes were detected. In addition, the NCBI and Genecard database were used to query gene functions and determine promising candidates.

### Quantitative trait loci enrichment analysis

We used a R package called GALLO [31,32] to perform QTL enrichment. We perform QTL enrichment analysis by using our significant SNPs about LMA in order to further explore the influence of these SNPs on LMA.

## RESULTS

### Phenotype analysis and heritability estimation

After QC, 35,426 SNPs with genotypes on 511 DLY pigs were finally retained for subsequent analyses. The descriptive statistics of LMA for the 511 pigs are listed in Table 1, according to which, the average was 73.82±9.33 (cm^2^). The univariate model was used to estimate the SNP-based heritability of the LMA trait. The phenotypic variance was categorized into the variance, explained by the genetic component and the residual variance comprising the genetic relationship matrix. The genome heritability of LMA was 0.35±0.096, indicating moderate heritability. The results were similar to those published earlier, suggesting the accuracy of our slaughter phenotype determination and that the genetic technology could effectively promote the genetic improvement of LMA.

### Single-locus genome-wide association studies for loin muscle area

**Figure 1:**
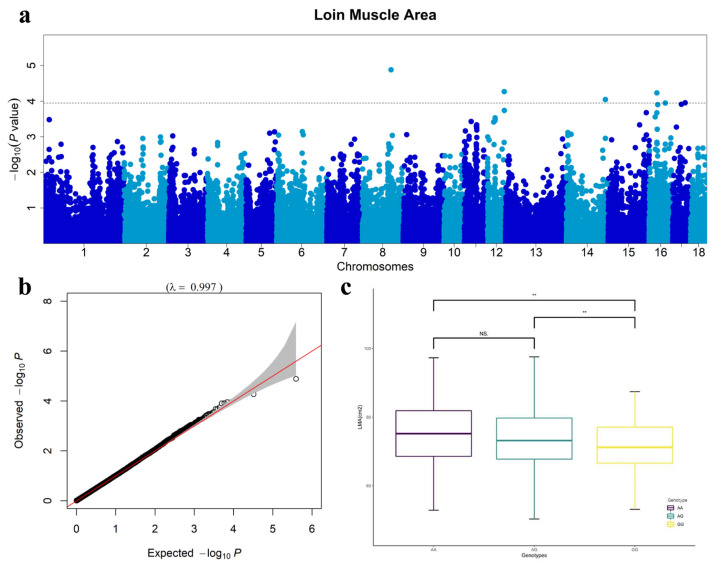
Manhattan, Quantile–quantile plots and boxplot. (a) Manhattan plot of genome-wide association studies (GWAS) result for loin muscle area (LMA). (b) Quantile–quantile (Q–Q) plot of genome-wide association study for LMA in the Duroc×(Landrace×Yorkshire) crossbred pigs (DLY) population. (c) Boxplot of genotypes of LMA. This boxplot presents the genotype frequencies for MARC0094338, and the counts of three genotypes (AA, AG, and GG) were 101, 278, and 132.

After QC using PLINK software, we obtained a total of 35,426 SNPs for subsequent analysis; then GEMMA software was used for GWAS analysis. Because Bonferroni correction is too conserved, it often leads to false negatives, and there may be few labeled association p-values in the whole genome that can meet this standard, hence, we used the FDR method. Finally, a total of six SNPs significantly related to LMA were identified in this study (Table 2). Moreover, the Manhattan plot of the GWAS result showed the significance of the association between 35,426 SNPs and LMA (Figure 1a). In the Manhattan plots, the -log10 p-values of the quantified SNPs were plotted against their genomic positions. *WU_10.2_8_104760632* located on Sus scrofa chromosome (SSC) 8, explained 0.65% of the phenotypic variance of LMA. *MARC 0094338*, located on SSC 12, accounted for 1.30% of the phenotypic variance of LMA, and *ASGA0072817*, located on SSC 16, accounted for 2.8% of the phenotypic variance of LMA. The other three SNPs (*WU_10.2_14_144250775*, *ALGA0090878*, and *ALGA0095241*) are located respectively on SSC14, SSC16, and SSC17 (Table 2). We picked the second most significant SNP (MARC0094338) as the top SNP, because there was no previous research on the most significant locus (WU_10.2_8_104760632) and it was not found in the NCBI database. For the SNP *MARC0094338*, 101 individuals with the AA genotype had higher LMA phenotype values than 278 pigs with the AG genotype and 132 with the GG genotype (Figure 1c). The frequencies of the three genotypes of *MARC0094338* (AA, AG, and GG) were 19.8%, 54.4%, and 25.8%, and the mean values of LMA were 74.57, 73.20, and 71.93 cm^2^. In summary, according to genotype counts and trends of MARC0094338s, the dominant allele frequency of this locus was not high in the current analysis, but it had a positive effect on LMA. This study also indicated that this locus could be continuously selected in the future to improve LMA; besides, the Q-Q plot did not show any sign of inflation (Figure 1b).

### Candidate gene search and functional analysis

According to the *Sus scrofa* 11.1 genome assembly, 11 functional genes were targeted (Table 2). Then, these genes were used to conduct KEGG pathways and GO analyses. In brief, the most significant pathway for LMA was related to the synthesis and degradation of ketone bodies (Supplementary Figure S1a). QTL enrichment analysis showed that meat, carcass, health, and exterior traits were mainly harbored in this pathway (Supplementary Figure S1b). The top GO term of LMA annotated genes were related to the regulation of histone acetylation and growth hormone receptor (GHR) signaling pathway. Given that numerous genes are involved in important pathways and biological processes, functional annotations mentioned in the NCBI database, and a number of earlier published studies were examined and verified; hence, two genes, including 3-oxoacid Co A-transferase 1 (*OXCT1*) and *GHR*, with biological functions such as increasing available amino acids in skeletal muscle, fat deposition, were selected as promising candidates for LMA [20].

## DISCUSSION

Proper assessment of pig-related indicators is the basis for determining the level of pig performance and growth-related traits. LMA is an influential trait of commercial pigs, affecting production performance, regarded as carcass traits, and plays essential roles in the determination of growth traits [33]. Compared to previous studies, we used cross-bred pigs, which have more complex genome than that of a purebred pig; these were subjected to strong positive selection, and the linkage disequilibrium of DLY crossbred was lower [34], and the genomic information obtained using different quality and density of sequencing chips also varied. In addition, various number of individuals and data filtering conditions also lead to differences in GWAS analyses. In pigs, the major genes specific to the LMA have not yet been identified, and our results are an addition to this area. For pig breeding in the future, it is necessary to clarify the genetic mechanism of LMA. Herein, we performed single-locus GWAS using 511 DLY individuals and identified six loci significantly associated with LMA. In the actual breeding for LMA selection, the SNPs results which we identified through GWAS can be taken as prior biological information into genomic models such as the genomic feature BLUP approach (GFBLUP) [35]. Moreover, recently, researchers proposed several new procedures for calculating SNP weights in wssGBLUP (an extension of ssGBLUP) that can be effective in improving both the accuracy of genomic estimated breeding values and SNP effects [36]. As far as we know, GWAS studies on LMA using DLY populations are still in the minority [37], and actual production could be better served using DLY for the GWAS of LMA. Understanding the genetic basis of phenotypic variation in DLY pigs will help to improve the effectiveness of purebred selection [34]. Other significant SNPs in chromosomes were found for the first time in the present study. This study contributes both to molecular breeding for DLY carcass traits and improves our knowledge of the loci or genes for LMA in commercial pigs.

The *GHR* gene located at the *ASGA0072817* locus of SSC 16 is found to have different degrees of influence on the expression of *GHR* in the longissimus dorsi and trapezius muscles due to moderate dietary restriction in adults [37], indicating that the *GHR* gene affects the growth of the longissimus dorsi muscle. The GHR is a single-chain, 620 amino acids transmembrane protein [37]. Hematopoietic, a member of the hematopoietic factor (Cytokine) receptor superfamily, is synthesized and secreted in animals by eosinophils in the anterior pituitary gland and plays a very crucial role in animal growth, development, reproduction, lactation, and immunity [38]. *GHR* studies in pigs revealed that the abundance of *GHR* mRNA increased in the longissimus dorsi during mild postpartum malnutrition. Litters were weaned at week 3, and muscle specific *GHR* expression was assessed for the next three weeks based on optimal or low food intake, resulting in the expression of the *GHR* gene and its regulation in mild malnutrition. GHR is related to metabolism, contraction, and specific functions of different muscles [39].

Another locus worthy of attention was *ASGA0072817*, located on SSC 16. The gene *OXCT1*, also known as the succinyl-CoA:3-ketoacid CoA transferase (*SCOT*) gene, located near this locus, encodes a key enzyme in ketone metabolism in the body and is also a catalytic switch for ketone decomposition and speed limit steps. In previous studies, such as in Chinese Holstein cows, *OXCT1* was identified as a new candidate gene that may affect milk fatty acid synthesis [40,41]. In the lactation period of the yak, the *OXCT1* gene was observed to be significantly up-regulated in the mammary gland tissue [41]. The *OXCT1* gene can also have a role in adipose tissue and adipocytes. In the adipose tissue of people with higher fat content, regardless of whether its metabolic function is normal or not, the expression of the *OXCT1* gene is lower than in people with lower body fat content [42]. In pigs, long non-coding RNAs are involved in important cellular activities in pigs, such as epigenetics, engraftment, and cell growth. Transcriptome analysis was performed in some studies on the back adipose tissue of Korean native pig×Yorkshire pig; KEGG analysis showed that *OXCT1* was significantly enriched and involved in the formation of fat [43].

Interestingly, for other genes near significant locus, although some studies have reported that they influence LMA or adipose muscle in pigs, others have reported that these genes are related to cancer or tumor diseases in human pathology. The myocardin (*MYOCD*) gene locus near the *MARC0094338* on SSC 12 is associated with lung cancer [44], the ARho GTPase activating protein 44 (*ARHGAP44*) gene is associated with tumors [45], the ElaC Ribonuclease Z 2 (*ELAC2*) gene is reported to be associated with prostate cancer [46], the mitogen-activated protein kinase kinase 4 (*MAP2K4*) gene is associated with breast cancer [47], F-Box protein 4 (*FBXO4*) located near *ASGA0072817* on SSC 16 is associated with cancer [48], and fibrillarin like 1 (*FBLL1*) located near WU_*10.2_14_144250775* on SSC 14 is associated with liver cancer [49]. In addition, the arginyl-TRNA synthetase 1 (*RARS1*) gene has been shown to be associated with various diseases of the central nervous system [50], slit guidance ligand 3 (*SLIT3*) located near *ALGA0090878* on SSC16 is associated with the bone loss [51], and the receptor activator of NF-Kb 3 (*RANK3*) gene is associated with alcoholic femoral head necrosis [52]. While these genes are currently poorly studied in pigs, but do not exclude the possibility of their association with LMA, the *OXCT1* gene has been well-documented to play a pivotal role in supporting malignancy [53], with studies in China pointing to its association with adipose development. The population used in this study was DLY ternary crossbred pigs, which has certain limitations in comparison with the reference genome [20]; thus, these genes have great potential to be discovered in the future.

## CONCLUSION

In this study, 35,426 SNPs from 511 DLY pigs were used for GWAS analysis. Six significant SNPs were associated with LMA, and two candidate genes (*GHR* and *OXCT1*) have been found that maybe influence LMA. In addition, other genes (*MYOCD*, *ARHGAP44*, *ELAC2*, *MAP2K4*, *FBXO4*, *FBLL1*, *RARS1*, *SLIT3*, and *RANK3*) are pending in the future. It is hoped that the results of this study can be used to determine the LMA of pigs and can provide a reference for the genetic improvement of traits.

## Figures and Tables

**Table 1:** Descriptive statistics and heritability for LMA

Traits	Number	Unit	Mean (±SD)	Min	Max	C.V./%	*h* ^2^ ^1)^
LMA	511	cm^2^	73.82±9.33	45.96	107.21	12.6	0.35±0.096

LMA, loin muscle area; N, number; SD, standard deviation; Min, minimum; Max, maximum; CV, coefficient of variation; *h*^2^, heritability.

1) Heritability (standard error) of LMA values.

**Table 2:** Description of SNPs significantly associated with LMA

Chromosome	SNPID	Position (bp)	EPV (%)	p-value	Gene^1)^
8	WU_10.2_8_104760632	97,865,933	0.6365	1.30E-05	-
12	MARC0094338	56,959,881	1.3021	5.39E-05	*ARHGAP44, ELAC2, MAP2K4*
16	ASGA0072817	26,426,528	2.8139	5.85E-05	*RIMOC1, FBX04,* ***GHR, OXCT1***
14	WU_10.2_14_144250775	132,664,263	1.4557	8.99E-05	-
17	ALGA0095241	43,212,985	1.8460	1.11E-04	*MAFB, FBLL1, RARS1*
16	ALGA0090878	54,945,912	0.9760	1.12E-04	*RANK3*

SNP, single nucleotide polymorphism; LMA, loin muscle area; EPV, the proportion of the phenotypic variance explained by significant SNP; *ARHGAP44*, ARho GTPase activating protein 44; *ELAC2*, ElaC Ribonuclease Z 2; *MAP2K4*, mitogen-activated protein kinase kinase 4; *RIMOC1*, RAB7A interacting MON1-CCZ1 complex Subunit 1; *FBX04*, F-Box protein 4; *GHR*, growth hormone receptor; *OXCT1*, 3-oxoacid Co A-transferase 1; *MAFB*, MAF BZIP transcription factor B; *FBLL1*, fibrillarin like 1; *RARS1*, arginyl-TRNA synthetase 1; *RANK3*, receptor activator of NF-Kb 3.

1) The bold genes in the table are candidate genes.

## SUPPLEMENTARY MATERIAL

**Supplementary Figure S1**. Kyoto encyclopedia of genes and genomes (KEGG) and gene ontology (GO) analysis.

**Supplementary Table S1**. p-values for fixed affects
