# Supplementary material for: Genome-wide association study for loin muscle area of commercial crossbred pigs
Source: Anim Biosci. 2023 Jan 11;36(6):861–8. doi: 10.5713/ab.22.0407 (PMC10164538; doi:10.5713/ab.22.0407)
Supplement: Supplementary file 1 [file ab-22-0407-Supplementary-Fig-1.pdf]

a

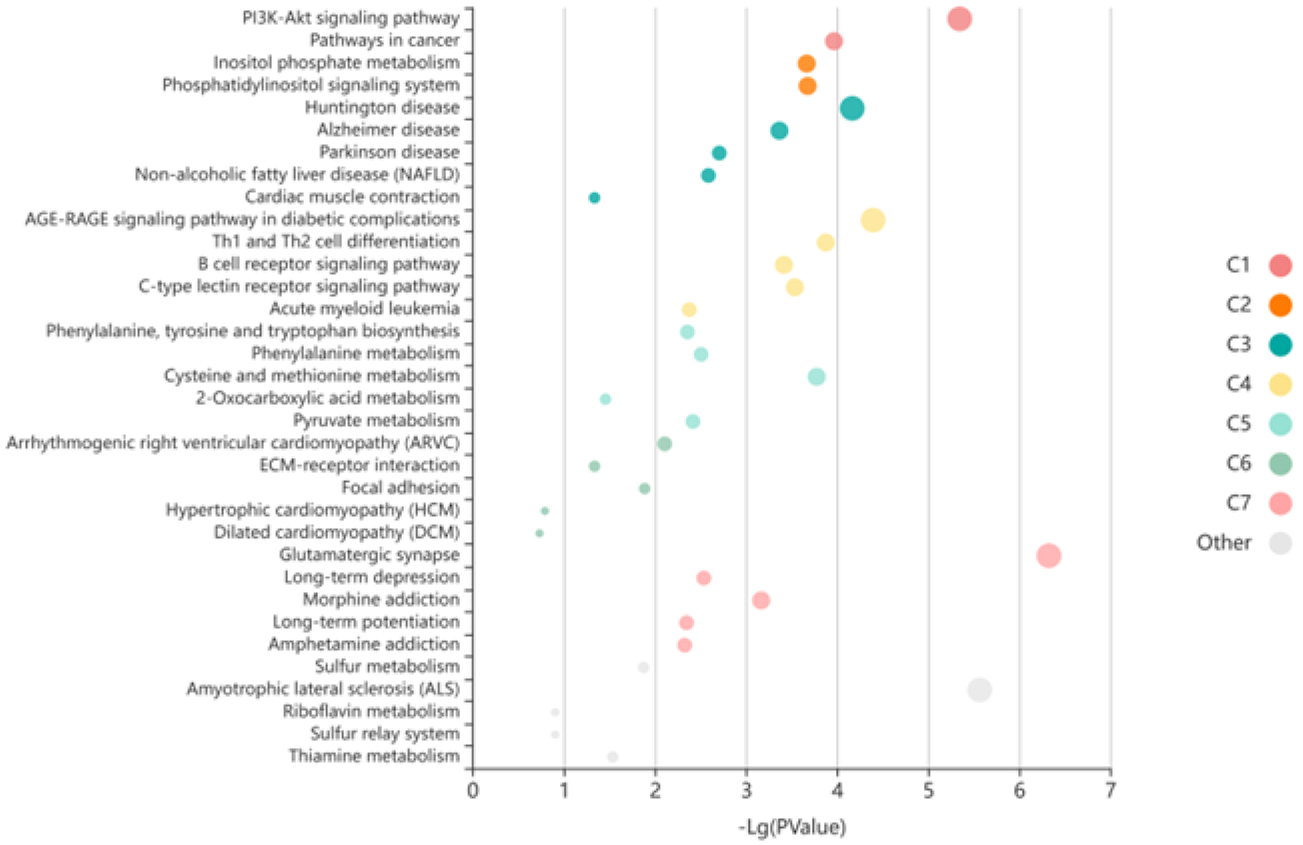
PI3K-Akt signaling pathway
Pathways in cancer
Inositol phosphate metabolism
Phosphatidylinositol signaling system
Huntington disease
Alzheimer disease
Parkinson disease
Non-alcoholic fatty liver disease (NAFLD)
Cardiac muscle contraction
AGE-RAGE signaling pathway in diabetic complications
Th1 and Th2 cell differentiation
B cell receptor signaling pathway
C-type lectin receptor signaling pathway
Acute myeloid leukemia
Phenylalanine, tyrosine and tryptophan biosynthesis
Phenylalanine metabolism
Cysteine and methionine metabolism
2-Oxocarboxylic acid metabolism
Pyruvate metabolism
Arrhythmogenic right ventricular cardiomyopathy (ARVC)
ECM-receptor interaction
Focal adhesion
Hypertrophic cardiomyopathy (HCM)
Dilated cardiomyopathy (DCM)
Glutamatergic synapse
Long-term depression
Morphine addiction
Long-term potentiation
Amphetamine addiction
Sulfur metabolism
Amyotrophic lateral sclerosis (ALS)
Riboflavin metabolism
Sulfur relay system
Thiamine metabolism
0
1
2
3
4
5
6
7
-Lg(PValue)
C1
C2
C3
C4
C5
C6
C7
Other

b

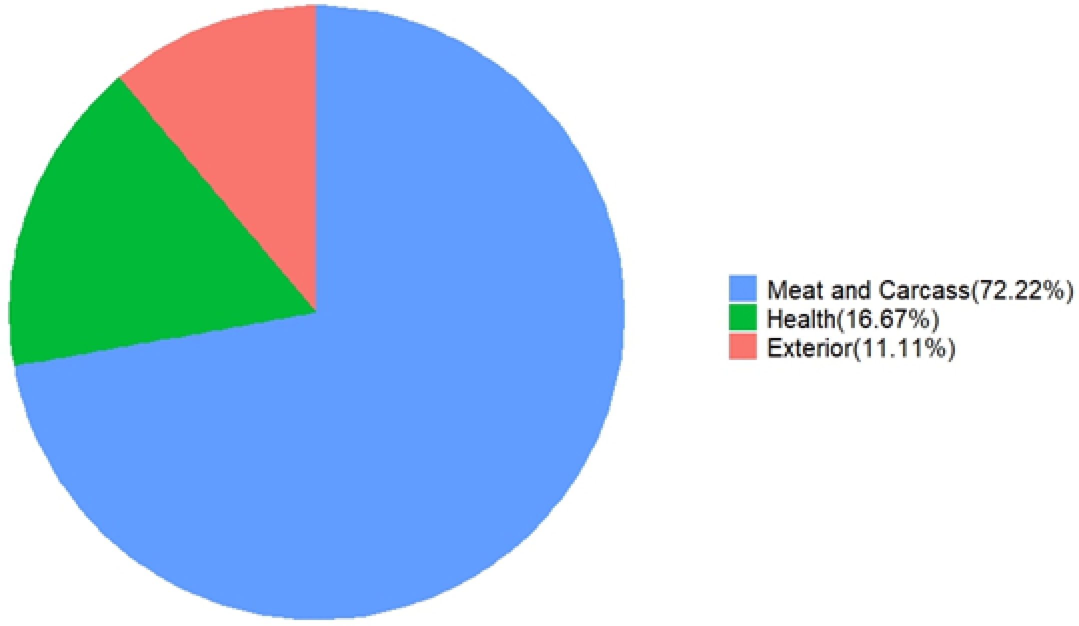
Meat and Carcass(72.22%)
Health(16.67%)
Exterior(11.11%)

Supplementary Material

Supplementary Figure S1. Kyoto encyclopedia of genes and genomes (KEGG) and gene ontology (GO) analysis. **a.** Significant GO terms and KEGG pathways for genes **b.** Qunatitative trait loci enriched results.

Supplementary Table S1. p-values for fixed affects

Fixed effects	PC1[b]	PC2[b]	PC3[b]	PC4[b]	PC5[b]	Slaughter_batch	Live_weight
p_value[1)]	0	$7.51175e^{-287}$	$1.34436e^{-175}$	$3.66312e^{-176}$	$5.52682e^{-174}$	$<2e^{-16}$	$<2e^{-16}$

[1)] p-value is calculated by EIGENSTRAT software and the R language b.PC1-PC5 are the five principal components are calculated using PLINK.
