# Supplementary material for: Genome-wide association study for loin muscle area of commercial crossbred pigs
Source: Anim Biosci. 2023 Jan 11;36(6):861–8. doi: 10.5713/ab.22.0407 (PMC10164538; doi:10.5713/ab.22.0407)
Supplement: Supplementary file 2 [file ab-22-0407-Supplementary-Table-1.pdf]

Supplementary Material

Supplementary Figure S1. Kyoto encyclopedia of genes and genomes (KEGG) and gene ontology (GO) analysis. **a.** Significant GO terms and KEGG pathways for genes **b.** Qunatitative trait loci enriched results.

Supplementary Table S1. p-values for fixed affects

Fixed effects	PC1[b]	PC2[b]	PC3[b]	PC4[b]	PC5[b]	Slaughter_batch	Live_weight
p_value[1)]	0	$7.51175e^{-287}$	$1.34436e^{-175}$	$3.66312e^{-176}$	$5.52682e^{-174}$	$<2e^{-16}$	$<2e^{-16}$

[1)] p-value is calculated by EIGENSTRAT software and the R language b.PC1-PC5 are the five principal components are calculated using PLINK.
